# Supplementary material for: In vivo localization of chronically implanted electrodes and optic fibers in mice
Source: Nat Commun. 2020 Sep 17;11:4686. doi: 10.1038/s41467-020-18472-y (PMC7499215; doi:10.1038/s41467-020-18472-y)
Supplement: Supplementary file 1 — Supplementary Information [file 41467_2020_18472_MOESM1_ESM.pdf]

In Vivo Localization of Chronically Implanted Electrodes and Optic Fibers in Mice

Király et al.

Supplemental Information

Supplemental figures

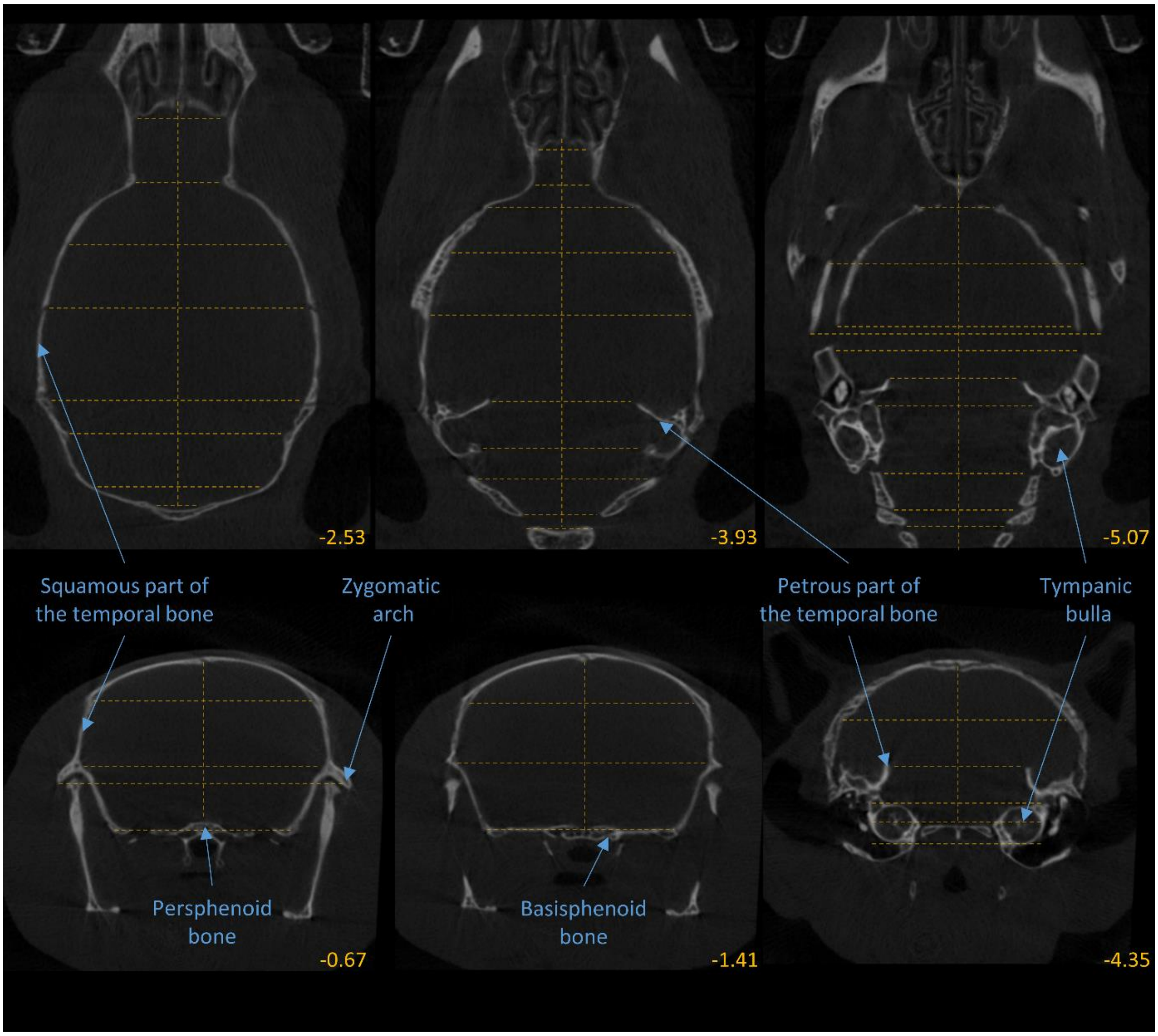

Supplementary Figure 1. Leveling the skull in the CT images. Horizontal (top) and coronal (bottom) images of the same animals' pre-operative CT scan at different levels (grayscale images; yellow numbers refer to the dorso-ventral and antero-posterior coordinates from Bregma in mm), aligned with the canonical 3D axes used in brain atlas coordinate systems by leveling symmetrical bone structures (blue arrows and yellow dashed lines).

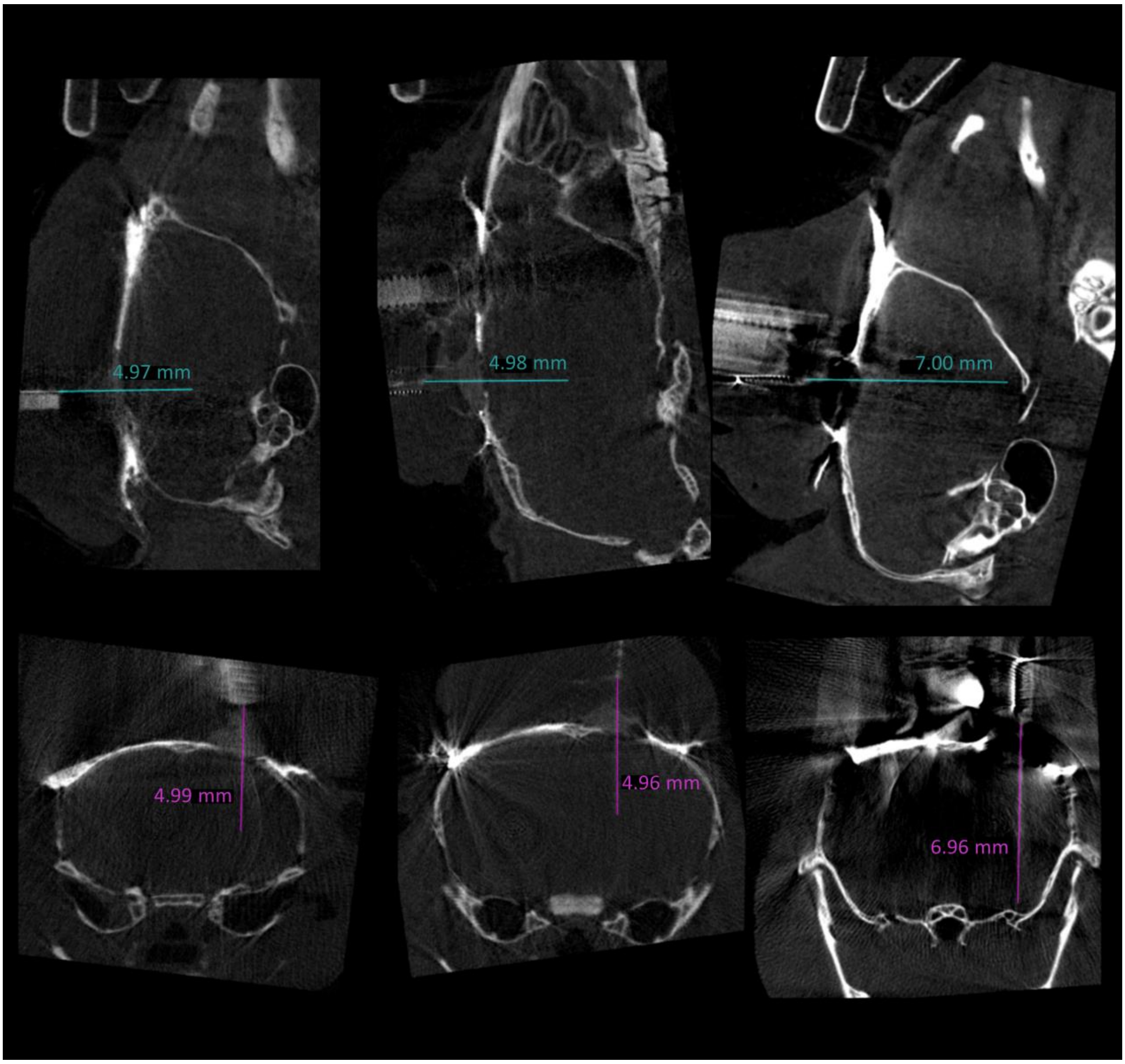

Supplementary Figure 2. Silicon probe length measurements. Sagittal (top) and coronal (bottom) post-operative CT scan (grayscale images) of 3 mice implanted with silicon probes. The total length of the probes from base to tip was read both from the sagittal (teal numbers) and coronal view images (purple numbers) and averaged. Measured lengths were compared with the nominal lengths of the probes (from left to right: 5mm, 5mm ,7mm).

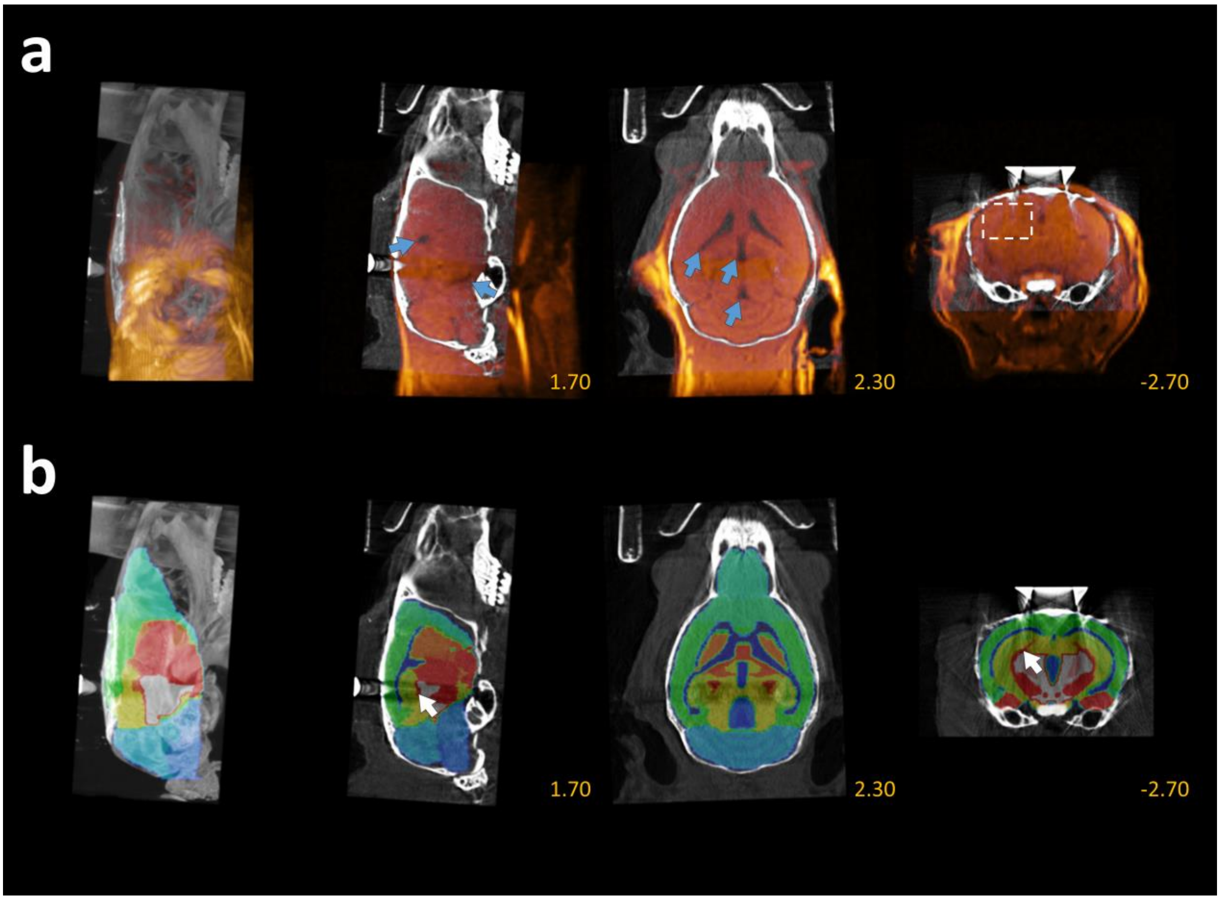

Supplementary Figure 3. CT-MRI fusion-based localization of an optic fiber targeting the hippocampus. **a** Post-operative CT image co-registered with the high SNR T_1-weighted pre-operative MRI image showing the hippocampus (white dashed box) in addition to the ventricles (blue arrows). **b** 3D atlas co-registered with the post-operative CT image using the CT-MRI fusion technique, paying special attention to the alignment of the atlas with the hippocampal structure. The white arrow shows the position of the optic fiber in the hippocampus. CT, MRI, and atlas images are shown with gray, gold and colored scales respectively. Images in both rows from left to right, maximum intensity projection, sagittal slice, horizontal slice, coronal slice. Yellow numbers refer to the corresponding stereotaxic coordinates of the plane relative to Bregma.

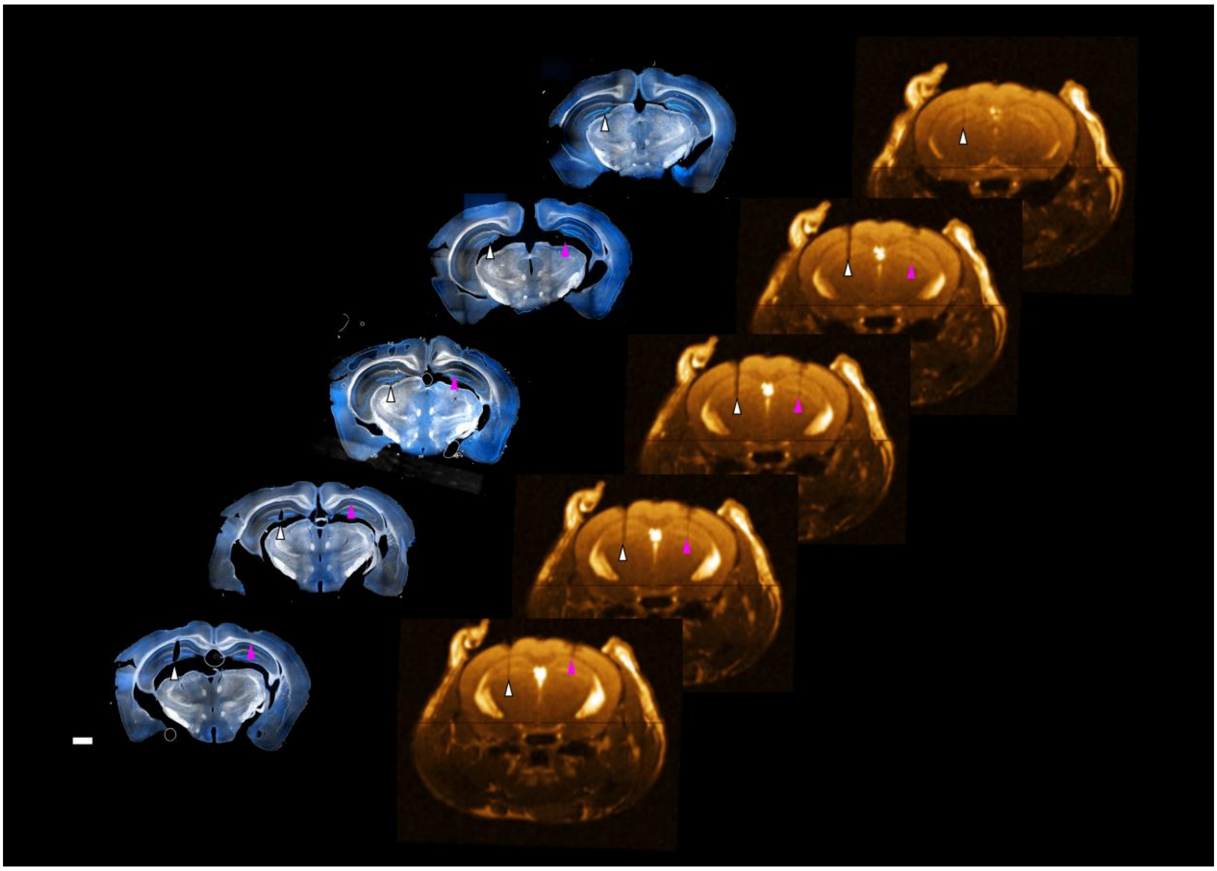

Supplementary Figure 4. Slice-by-slice comparison of *in vivo* post-operative MRI and histology. T_2/T_1-weighted MRI images (right, gold scale images) were compared with the corresponding histological slices (left, dark-field images overlaid with fluorescent DAPI images) for the animal not presented in Fig. 6b, showing the track of a 50 µm (magenta arrows, left hemisphere) and a 105 µm (white arrows, right hemisphere) core diameter optic fiber. Scale bar, 1 mm.

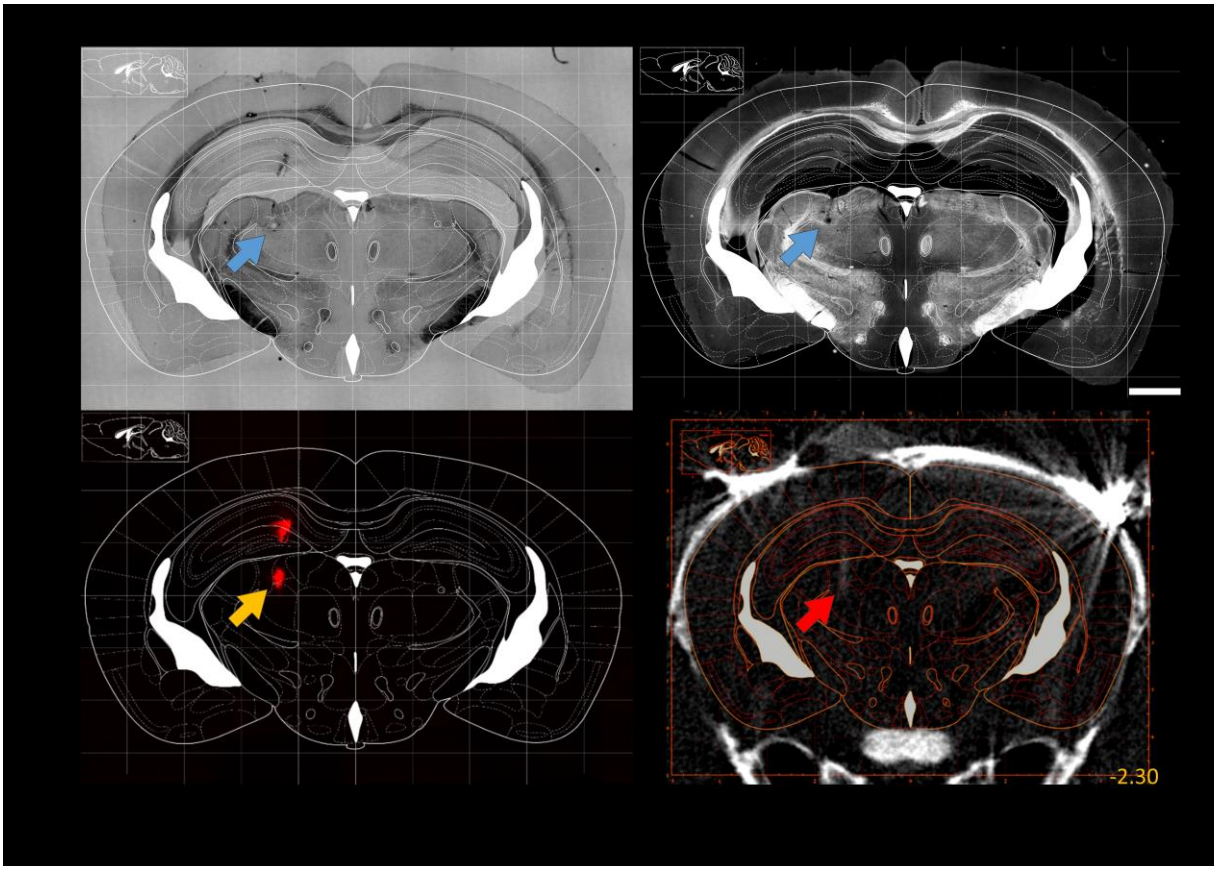

Supplementary Figure 5. Silicon probe track reconstructed with histology and in vivo. The Paxinos atlas was fitted on coronal sections based on anatomical landmarks identified in the bright-field (top left) and dark-field (top right) images. The atlas image was overlaid on fluorescent images showing the implant track (DiI, yellow arrow, bottom left). The tip of the probe was marked with an electrolytic lesion (blue arrows). The corresponding coronal CT slice (grayscale image) with the implant trajectory localized in vivo (red arrow, bottom right) was compared to the histologically reconstructed track. Yellow number refers to the antero-posterior coordinate of the plane relative to Bregma. Scale bar, 1 mm.

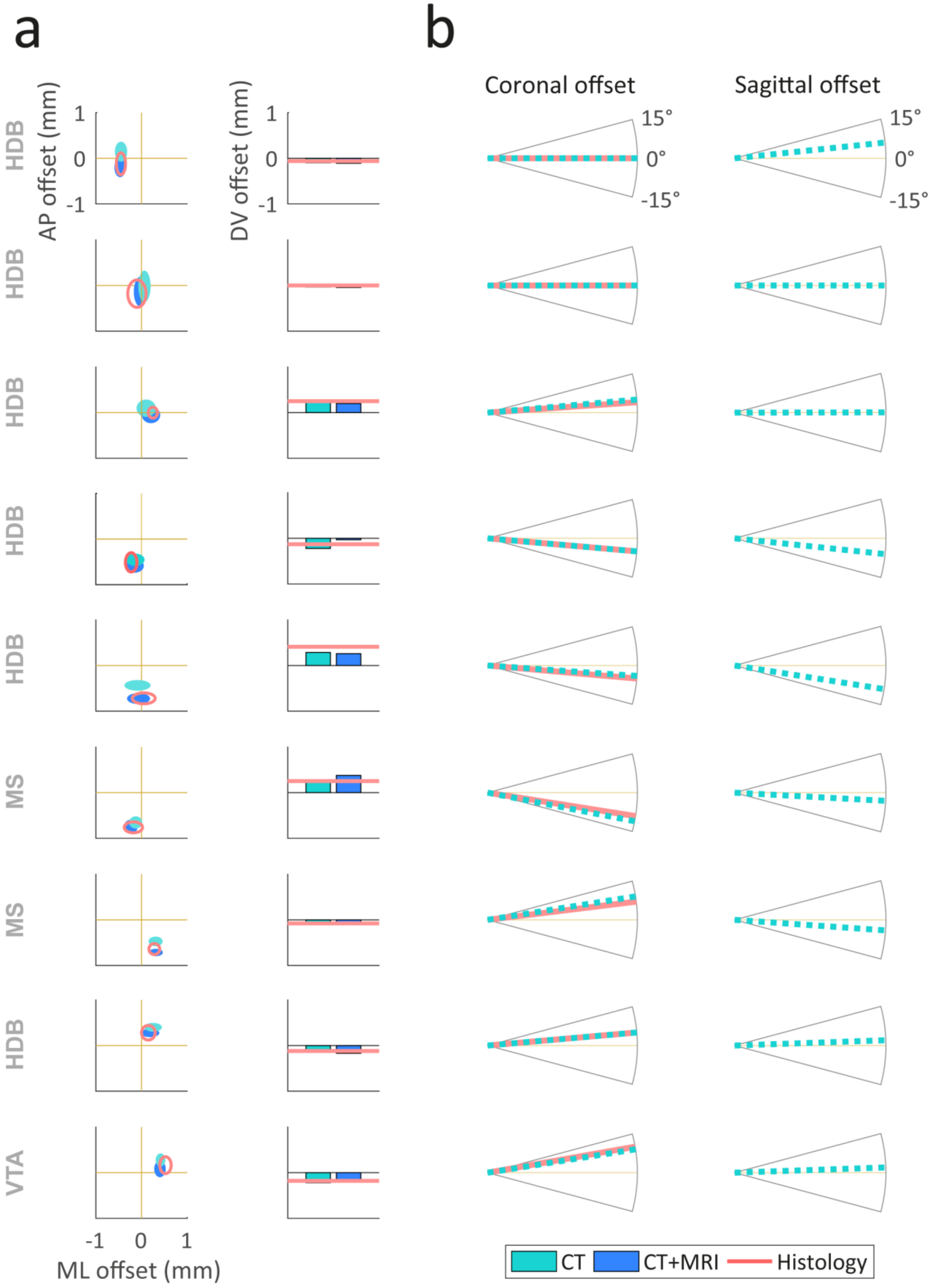

Supplementary Figure 6. Quantification of localization accuracy for tetrode implants targeting deep brain targets. a Offset from the antero-posterior (AP), medio-lateral (ML) and dorso-ventral (DV) target

coordinates for all mice. Teal, CT; blue, CT-MRI fusion; salmon, histology. Ellipses represent the area covered by the tip of the implant in the horizontal plane. **b** Coronal and sagittal offset from the planned direction of trajectory. Implant directions were quantified based on the CT images. Histology only provides coronal offset measures. Anterior, lateral and ventral directions were defined as the positive directions. Source data are provided as a Source Data file.

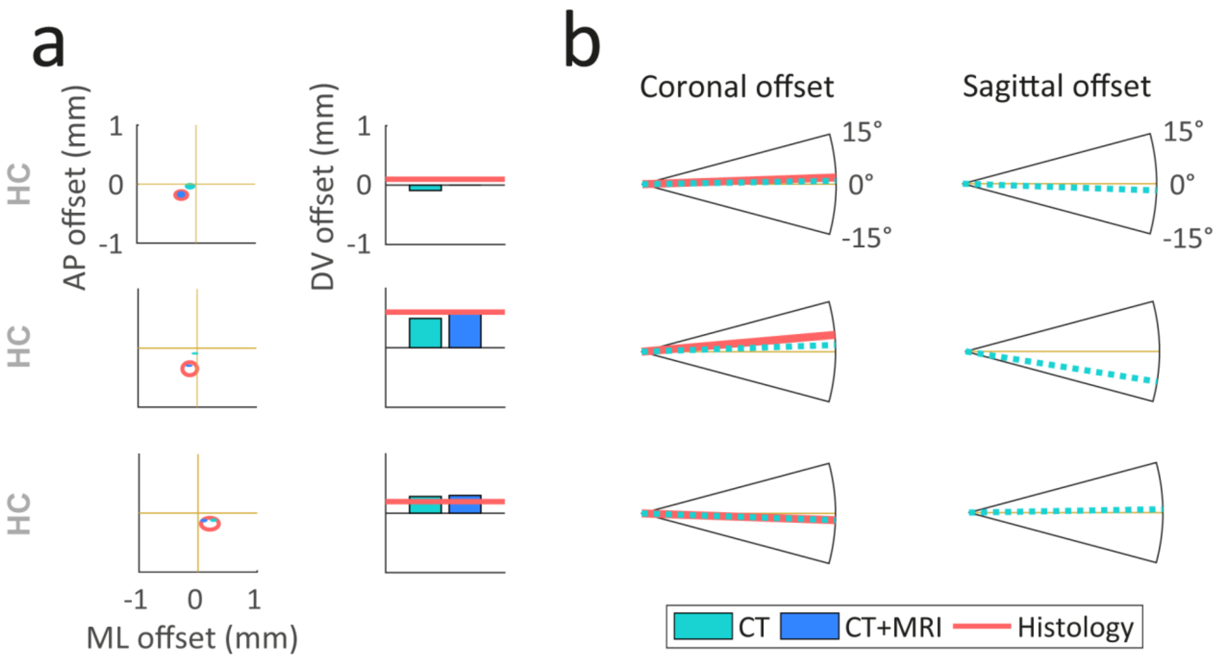

Supplementary Figure 7. Quantification of localization accuracy for optic fiber and silicon probe implants targeting the hippocampus (HC). a Offset from the antero-posterior (AP), medio-lateral (ML) and dorso-ventral (DV) target coordinates for all mice. Teal, CT; blue, CT-MRI fusion; salmon, histology. Ellipses represent the area covered by the tip of the implant in the horizontal plane (optic fiber in the top row, silicon probes in the middle and the bottom rows). **b** Coronal and sagittal offset from the planned direction of trajectory. Implant directions were quantified based on the CT images. Histology only provides coronal offset measures. Anterior, lateral and ventral directions were defined as the positive directions. Source data are provided as a Source Data file.

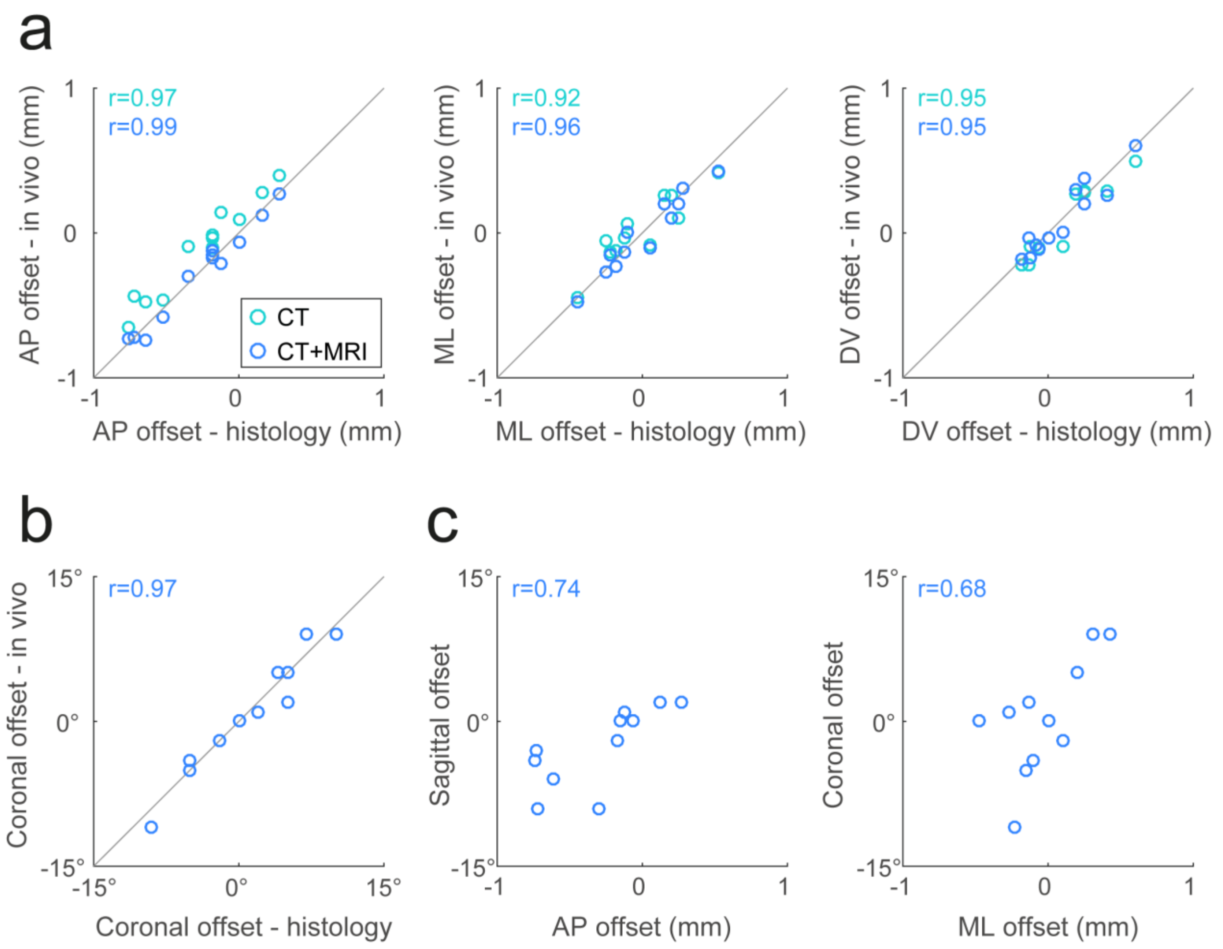

Supplementary Figure 8. Correlation between offset measures. a Antero-posterior (AP), medio-lateral (ML), dorso-ventral (DV) offsets from target measured in vivo (teal, CT; blue, CT-MRI fusion) vs. by histology (n = 12 implants). **b** Coronal implant direction measured in vivo vs. by histology (n = 12 implants). **c** AP offsets vs. sagittal direction offsets (n = 11 implants) and ML offsets vs. coronal direction offsets (n = 12 implants) measured with the CT-MRI fusion. r, Pearson's correlation coefficient. Source data are provided as a Source Data file.

Setting code	(i)	(ii)	(iii)	(iv)	(v)
Magnification (resolution)	2.47 (19 µm)	2.47 (19 µm)	2.47 (19 µm)	1.36 (35 µm)	1.36 (35 µm)
Number of projections	360	360	180	360	180
Photon energy (kVp)	45	45	45	45	45
Exposure time (ms)	1300	500	500	500	500

Silicone probe

Optic fiber

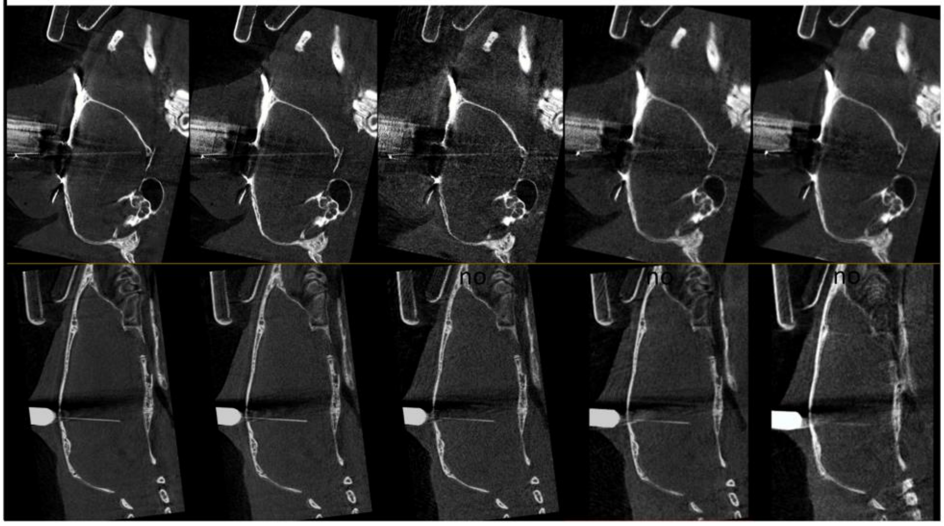

Supplementary Figure 9. Optimization of image quality for optic fibers and silicon probes. CT images (grayscale) acquired with different settings (top) of a mouse implanted with a silicon probe (Buzsaki-type, middle) and one with an optic fiber (105 µm core diameter, bottom).

Supplemental Tables

Implant type	Dimensions
tetrode wire	12.7 µm diameter
8 tetrode bundle (custom built)	approximately cylindrical volume (with gaps between tetrodes) with an average diameter of 340 ± 70 µm
optic fiber	105 µm core diameter, 250 µm outer diameter
optic fiber	50 µm core diameter, 65 µm outer diameter
Buzsaki-type probe	52 × 15 µm single shank; length: 7 mm
polytrode probe	113 x 15 µm single shank; length: 5 mm
edge probe	150 x 15 µm single shank; length: 5 mm

Supplementary Table 1. Dimensions of different implant types. The average size (± standard deviation) of the tetrode bundles is based on the CT images.

Brain area	AP Coordinate (mm)	ML Coordinate (mm)	DV Coordinate (mm)
HDB (tetrode, optic fiber)	0.74	0.6	5.0
MS (tetrode)	0.9	0.1	3.9
VTA (tetrode, optic fiber)	-3.1	0.6	4.0
ventral hippocampus (Buzsaki-type probe)	-2.5	2	4.7
dorsal hippocampus (polytrode probe)	-2	1.5	2.3
dorsal hippocampus (edge probe)	-3	2	2.5
dorsal hippocampus (optic fiber)	-2.5	2	2.3

Supplementary Table 2. Target coordinates for implantation surgeries. Numbers are atlas coordinates in mm from Bregma. AP, antero-posterior; ML, medio-lateral; DV, dorso-ventral.
