# Supplementary material for: In vivo localization of chronically implanted electrodes and optic fibers in mice
Source: Nat Commun. 2020 Sep 17;11:4686. doi: 10.1038/s41467-020-18472-y (PMC7499215; doi:10.1038/s41467-020-18472-y)
Supplement: Supplementary file 3 — Reporting Summary [file 41467_2020_18472_MOESM3_ESM.pdf]

nature research

Corresponding author(s): Balazs Hangya

Last updated by author(s): Jul 20, 2020

Reporting Summary

Nature Research wishes to improve the reproducibility of the work that we publish. This form provides structure for consistency and transparency in reporting. For further information on Nature Research policies, see our Editorial Policies and the Editorial Policy Checklist.

Statistics

For all statistical analyses, confirm that the following items are present in the figure legend, table legend, main text, or Methods section.

n/a	Confirmed	
☐	☒	The exact sample size (*n*) for each experimental group/condition, given as a discrete number and unit of measurement
☒	☐	A statement on whether measurements were taken from distinct samples or whether the same sample was measured repeatedly
☐	☒	The statistical test(s) used AND whether they are one- or two-sided<br>*Only common tests should be described solely by name; describe more complex techniques in the Methods section.*
☒	☐	A description of all covariates tested
☐	☒	A description of any assumptions or corrections, such as tests of normality and adjustment for multiple comparisons
☐	☒	A full description of the statistical parameters including central tendency (e.g. means) or other basic estimates (e.g. regression coefficient) AND variation (e.g. standard deviation) or associated estimates of uncertainty (e.g. confidence intervals)
☐	☒	For null hypothesis testing, the test statistic (e.g. *F*, *t*, *r*) with confidence intervals, effect sizes, degrees of freedom and *P* value noted<br>*Give P values as exact values whenever suitable.*
☒	☐	For Bayesian analysis, information on the choice of priors and Markov chain Monte Carlo settings
☒	☐	For hierarchical and complex designs, identification of the appropriate level for tests and full reporting of outcomes
☐	☒	Estimates of effect sizes (e.g. Cohen's *d*, Pearson's *r*), indicating how they were calculated

Our web collection on statistics for biologists contains articles on many of the points above.

Software and code

Policy information about availability of computer code

Data collection	Nucline 2.01 was used for scanning.
Data analysis	Analyses were carried out in VivoQuant 1.22 (inviCRO, Boston, MA, USA) and Matlab R2016a (Mathworks, Natick, MA, USA).

For manuscripts utilizing custom algorithms or software that are central to the research but not yet described in published literature, software must be made available to editors and reviewers. We strongly encourage code deposition in a community repository (e.g. GitHub). See the Nature Research guidelines for submitting code & software for further information.

Data

Policy information about availability of data

All manuscripts must include a data availability statement. This statement should provide the following information, where applicable:

- Accession codes, unique identifiers, or web links for publicly available datasets
- A list of figures that have associated raw data
- A description of any restrictions on data availability

Co-registered micro-CT and MRI images were made available via Figshare (https://doi.org/10.6084/m9.figshare.12700997.v3). Source data are provided with this paper. Specifically, The source data underlying Figures 8 and 10 and Supplementary Figures 6, 7 and 8 are provided as source data files.

Field-specific reporting

Life sciences study design

All studies must disclose on these points even when the disclosure is negative.

Sample size	No statistical methods were used to pre-determine sample size. However, sample size was chosen based on other studies of the field, most importantly Rangarajan et al., 2016.
Data exclusions	One animal with a bent implant (Fig. 9a) was excluded from a subset of analyses (noted in the text; Fig. 8g and Supplementary Fig. 9c) because it would have distorted the estimations of systematical implantation errors during the surgical procedure.
Replication	During revision of this study, we performed new experiments to localize hippocampal implants and increased the number of animals involved in the behavioral test. Both experiments replicated our previous findings regarding the quantification of localization accuracy and the absence of adverse effect of the scanning protocol on behavior. The localization procedure combining CT and MRI imaging was performed independently for n = 12 implantations involving three different implant types (tetrodes, silicon probes, optic fibers) and targeting four different brain areas (HDB, VTA, MS, hippocampus). In addition to this, independent CT based localization was performed for two optic fiber and a silicon probe implant and two optic fiber implants were localized based on post-operative MRI scans. Implants could be reproducibly localized in all of these experiments, as demonstrated in the figures.
Randomization	For the behavioral test, 7 out of 14 animals were randomly allocated into the test group. Behavioral trials were presented in randomized order. Other parts of this study did not involve separate experimental groups.
Blinding	Pre-operative behavioral training was performed in a fully automated system without any interaction with the experimenters (Birtalan et al., bioRxiv, 2020). Automated post-operative training was performed with minimal interactions, limited to transferring the animal from the home cage to the training chamber. Analysis of behavioral performance was fully automated. Experimenters were not blinded to group allocation, since the experiments were automated.

Reporting for specific materials, systems and methods

We require information from authors about some types of materials, experimental systems and methods used in many studies. Here, indicate whether each material, system or method listed is relevant to your study. If you are not sure if a list item applies to your research, read the appropriate section before selecting a response.

Materials & experimental systems

n/a	Involved in the study	
☒	☐	Antibodies
☒	☐	Eukaryotic cell lines
☒	☐	Palaeontology and archaeology
☐	☒	Animals and other organisms
☒	☐	Human research participants
☒	☐	Clinical data
☒	☐	Dual use research of concern

Methods

n/a	Involved in the study	
☒	☐	ChIP-seq
☒	☐	Flow cytometry
☐	☒	MRI-based neuroimaging

Animals and other organisms

Policy information about studies involving animals; ARRIVE guidelines recommended for reporting animal research

Laboratory animals	Wild type (n = 24) and genetically modified (ChAT-Cre, n = 22; ChAT-Cre × DAT-Cre, n = 4, DAT-Cre, n = 5) adult (3-4 months old, 25-30 g weight) male mice of C57BL/6N genetical background were used. Animals were housed individually in 36 × 20 × 15 cm cages under a standard 12-hours light-dark cycle (lights on at 8 a.m.) with food and water available ad libitum. Temperature and humidity were kept at 21 ± 1 °C and 50-60 %, respectively.
Wild animals	The study did not involve the use of wild animals.
Field-collected samples	The study did not involve samples collected from the field.
Ethics oversight	All experiments were approved by the Institutional Animal Care and Use Committee and the Committee for Scientific Ethics of Animal Research of the National Food Chain Safety Office (PE/EA/675-4/2016, PE/EA/1212-5/2017, PE/EA/864-7/2019) and were performed according to the guidelines of the institutional ethical code and the Hungarian Act of Animal Care and Experimentation (1998; XXVIII, section 243/1998, renewed in 40/2013) in accordance with the European Directive 86/609/CEE and modified according to the Directives 2010/63/EU. BK, IH, KSz and BH each hold a certificate course diploma of Advanced Radiation Protection (with certification numbers H04/2017, B-2019/54, OSSKI-2014-ÁK-369-21, SUVE-B-059/2008), granted by the Budapest University of Technology and Economics or the Semmelweis University, Budapest according to the Hungarian Act CXVI of 1996 on Atomic Energy ('Atomic Act'), granted based on the permission of the National Public Health Service of Hungary.

Note that full information on the approval of the study protocol must also be provided in the manuscript.

Magnetic resonance imaging

Experimental design

Design type

Structural mouse brain imaging, without behavioral task.

Design specifications

Isoflurane gas was used as an inhalation anesthetic through a specialized isoflurane mask, which was also used for head fixation to avoid motion artifacts. Magnetic resonance imaging was performed with a nanoScan PET/MRI system, which is equipped with a permanent magnetic field of 1 T and with a 450 mT/m gradient system using a volume coil for both reception and transmission. Fast T1-weighted images were acquired with a 3D gradient echo sequence using 8 excitations, TR = 15 ms repetition and TE = 2.2 ms echo times and 25° flip angle with resolution set to 0.28 mm. The sequence parameters were selected in order to achieve proper contrast and SNR that facilitates good visualization of the contour of the brain and the position of the ventricles with sufficient resolution in reasonable acquisition time (10 minutes). Translational-field magnetic resonance imaging was performed with a nanoScan 3T MRI system with a 3 T cryogen-free superconducting magnet and with a 600 mT/m gradient system using a 72 mm volume coil for transmission and a 20 mm surface coil for signal reception. High SNR T1-weighted images were acquired with a 3D gradient echo sequence using 3 excitations, TR = 25 ms repetition and TE = 2.7 ms echo times and 30° flip angle with resolution set to 0.2 mm, which enabled us to visualize the hippocampal formation with 20 minutes acquisition time. We also performed post-operative MRI imaging with the 3 T system. High resolution T2/T1-weighted 3D balanced steady-state free precession (bSSFP) sequence was performed using 12 excitations, TR = 5.13 ms repetition and TE = 2.565 ms echo times and 40° flip angle with resolution set to 0.12×0.12×0.15 mm. High resolution T2-weighted 2D fast spin echo sequence was performed using 20 excitations, TR = 3000 ms repetition and TE = 98.9 ms echo times with resolution set to 0.1×0.1×0.6 mm. The sequence parameters were set to achieve the best resolution and image quality, suitable for visualizing the track of both implants in the hippocampal formation, in reasonable acquisition time (20 minutes).

Behavioral performance measures

Structural imaging was performed without behavioral task.

Acquisition

Imaging type(s)

Structural imaging

Field strength

1 T and 3 T

Sequence & imaging parameters

- 3D gradient echo sequence using 8 excitations, TR = 15 ms repetition and TE = 2.2 ms echo times and 25° flip angle
- 3D gradient echo sequence using 3 excitations, TR = 25 ms repetition and TE = 2.7 ms echo times and 30° flip angle
- 3D balanced steady-state free precession (bSSFP) sequence using 12 excitations, TR = 5.13 ms repetition and TE = 2.565 ms echo times and 40° flip angle
- 2D fast spin echo sequence using 20 excitations, TR = 3000 ms repetition and TE = 98.9 ms echo times

Area of acquisition

whole brain

Diffusion MRI ☐ Used ☒ Not used

Preprocessing

Preprocessing software

VivoQuant 1.22 (inviCRO, Boston, MA, USA) was used for the co-registration of structural MRI images with CT images of the same animal and with the three-dimensional mouse brain atlas.

Normalization

Euclidean transformations (translations and rotations) and non-Euclidean affine transformations (only in step (iii) of the CT-MRI-atlas co-registration method) were used for CT-MRI-atlas co-registration.

Normalization template

Three-dimensional mouse brain atlas by Bai and colleagues:
Bai, J., Trinh, T. L. H., Chuang, K.-H. & Qiu, A. Atlas-based automatic mouse brain image segmentation revisited: model complexity vs. image registration. Magn. Reson. Imaging 30, 789–798 (2012).

Noise and artifact removal

No noise and artifact removal algorithm was used.

Volume censoring

No volume censoring was applied.

Statistical modeling & inference

Model type and settings

Structural MRI data was analyzed without modeling.

Effect(s) tested

Comparison between the results of histological localization and CT-MRI based in vivo localization were performed. Correlations were tested using Pearson's correlation coefficient (r), at a significance level of 0.05. The error measures were tested for statistical significance by the non-parametric two-tailed Wilcoxon signed rank test.

Specify type of analysis: ☒ Whole brain ☐ ROI-based ☐ Both

Statistic type for inference
(See Eklund et al. 2016)

This study used structural MRI scans (fMRI was not performed).
Systematic deviations from the target coordinate and directions were tested with CT-MRI based in vivo localization.

One-tailed t-tests were used, since approximately normal distribution could be reasonably assumed (normality was tested with and not rejected by Lilliefors-test, $p > 0.05$) and the null-hypothesis was one-sided for these tests.

Correction

Voxel-based statistical comparisons were not performed. Statistical comparisons of implant location and target are reported with exact p values, without correction.

Models & analysis

n/a	Involved in the study
☒	☐ Functional and/or effective connectivity
☒	☐ Graph analysis
☒	☐ Multivariate modeling or predictive analysis
